# Cardiomyopathy characterizing and heart failure risk predicting by echocardiography and pathoanatomy in aged male mice

**DOI:** 10.14814/phy2.70061

**Published:** 2024-10-16

**Authors:** Xiao‐Jun Du, Xin‐Heng Feng, Zi‐Qiu Ming, Helen Kiriazis

**Affiliations:** ^1^ Department of Physiology and Pathophysiology, School of Basic Medical Sciences Xi'an Jiaotong University Health Science Center Xi'an Shaanxi China; ^2^ Experimental Cardiology Baker Heart and Diabetes Institute Melbourne Victoria Australia; ^3^ Present address: Department of Cardiology and Institute of Vascular Medicine Peking University Third Hospital Beijing China; ^4^ Present address: Dandenong Neurology Dandenong Victoria Australia

**Keywords:** dilated cardiomyopathy, echocardiography, heart failure, risk prediction, transgenic mice

## Abstract

Correlation between echocardiographic and pathoanatomic variables and their prognostic value in murine cardiomyopathy models remain unknown. Using echocardiography, morphometrics, and survival monitoring, we characterized transgenic (TG) mice with dilated cardiomyopathy due to cardiac overexpression of β_2_‐adrenoceptors focusing on predicting heart failure (HF) risk and HF mortality. In 12‐month‐old non‐TG and TG mice, echocardiography was performed to determine left ventricular (LV) dimensions (d), wall thickness (h), and fractional shortening (FS). Animals were monitored for 3 months for survival. Organ weights and pathological events indicating left HF were determined. TG mice (*n* = 76) had reduced FS and enlarged LV, and 79% died of HF or likely arrhythmias during the follow‐up period while all non‐TG mice (*n* = 26) survived. These mice with left HF also had pulmonary congestion and hypertrophy/dilatation of the right ventricle (RV). Weights of lungs, RV, and atria were intercorrelated (*r* = 0.79–0.83) and also negatively correlated with FS × (*h*/*d*) index (*r* = −0.502 to −0.609). By FS × (*h*/*d*) tertiles, TG mice of low tertiles were identified with the highest mortality (96%) largely due to HF (76%). In conclusion, in aged cardiomyopathy mice a good correlation existed between echocardiographic and pathoanatomic variables. Echocardiography‐derived LV function and remodeling were useful in identifying a subgroup of TG mice with a high risk of HF and HF fatality.

## Graphical abstract

In aged TG mice, sustained activation of cardiac β‐adrenergic signaling results in progressive development of DCM. At its terminal phase, diversity of cardiophenotype becomes evident with TG mice showing a range of degrees of LV dysfunction and remodeling, RV hypertrophy and dilatation, and premature deaths due to arrhythmias or HF. The FS × (*h*/*d*) index allows for identifying a cohort of mice with high risk of HF and HF death together with right heart overload secondary to pulmonary congestion.

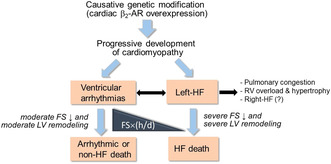

## 1 INTRODUCTION

Elderly human patients with symptomatic cardiomyopathy have a poor prognosis with a 5‐year survival of approximately 50%, and ventricular tachyarrhythmias and heart failure (HF) constitute the major causes of death (Cannata et al., 2020; Weintraub et al., 2017). Echocardiography is the most commonly used method for diagnosis, monitoring, and risk stratification of HF patients including a selection of candidates for nondrug therapies including ventricular assist devices or heart transplantation (Sinagra et al., 2020; Thomas et al., 2009). Recent studies have revealed that the presence of right heart dysfunction and failure (right HF) forms an independent risk factor for cardiomyopathy patients (Charisopoulou et al., 2019; Hiemstra et al., 2019; Tjahjadi et al., 2022).

By manipulating the murine genome, numerous mouse models of cardiomyopathy have been generated with phenotypes of HF, arrhythmias, and premature cardiac deaths, mimicking clinical settings (Du, 2001; Maass & Leinwand, 2000; Ross Jr., 2002). Among them are transgenic (TG) models with cardiac overexpression of β‐adrenergic receptors (β‐AR) that simulate the enhanced cardiac sympatho‐adrenergic activity well described in HF patients (Du, 2021). Studies by this and other groups have demonstrated that cardiac overexpression of β_1_‐AR, β_2_‐AR, stimulatory G‐protein, or protein kinase‐A, all resulted in similar DCM phenotypes (Dorn 2nd et al., 1999; Du, 2001; Du, Gao, Wang, et al., 2000; Engelhardt et al., 1999), implying an adverse class effect of enhanced β‐adrenergic signaling. We previously reported in β_2_‐TG mice the age‐dependent onset of dilated cardiomyopathy (DCM) leading to premature death (Du, Gao, Wang, et al., 2000). Features of the heart consist of cardiomegaly, extensive fibrosis, progressive cardiomyocyte dropout or hypertrophy (Du, Gao, Wang, et al., 2000; Lee et al., 2013), downregulation of genes of mitochondrial metabolism (Du, 2021), enhanced reactive oxidative stress and inflammatory signaling (Xu et al., 2011). Echocardiography or telemetry revealed in β_2_‐TG mice significant and age‐dependent LV remodeling and dysfunction, and spontaneous ventricular arrhythmias that are partially responsible for premature fatality (Du, Gao, Wang, et al., 2000; Lee et al., 2013; Nguyen et al., 2015; Xu et al., 2011).

While simulating the symptoms and histopathology that seen in clinical patients with cardiomyopathy, this β_2_‐TG strain exhibits certain diversities in cardiac phenotypes. Significant sex differences in cardiac physiology, histopathology, and survival with female advantages over male mice (Gao et al., 2003). Some male TG mice showed significant LV hypertrophy, whereas others degenerated into DCM. TG mice also differ in the mode of premature death due either to congestive HF or non‐HF reasons most likely lethal arrhythmias (Gao et al., 2003; Nguyen et al., 2015). While echocardiography is a routine tool in research on mouse cardiomyopathy models, there has been no report of prognostic prediction using echocardiography in mice. It also remains unknown whether echocardiographic parameters of mice with cardiomyopathy correlate with pathoanatomic abnormalities, the information unlikely to be obtained in the clinical setting.

The aims of the current study were to examine whether a single echocardiographic examination allows for predicting the outcome of aged β_2_‐TG mice. We used the TG strain of male mice that carry the identical β_2_‐AR transgene but show differences in the severity of DCM and HF, and the mode of premature deaths (Du, Gao, Wang, et al., 2000; Gao et al., 2003; Nguyen et al., 2015). Specifically, we aimed to address two questions: first, the relationship between echocardiographic and pathoanatomic variables in assessing the severity of cardiomyopathy; second, the usefulness of routine echocardiography in predicting the risk of HF and HF death.

## 2 MATERIALS AND METHODS

### 2.1 Animals

The β2‐AR TG strain was generated by Milano et al. (1994) and generously provided by Dr. Robert Lefkowitz in 1995. Its cardiomyopathy phenotype has been well defined by our previous studies (Du, Gao, Jennings, et al., 2000; Du, Gao, Wang, et al., 2000; Lee et al., 2013; Nguyen et al., 2015; Xu et al., 2011). TG mice were on a genetic background of C57BL/6J, and the genotype was screened by PCR testing of DNA extracted from tail biopsy. Animals were housed at an ambient room temperature of 22°C in an air‐conditioned facility with a 12/12 hour light‐dark cycle. Optimice® standard cage system was used with 2–4 mice per cage and bedding was changed weekly. Animals were provided with standard rodent chow (LabDiet®) and water at liberty. Our previous studies have shown significant sex differences in the cardiomyopathy phenotype with females exhibiting much less severe abnormalities (Gao et al., 2003), and therefore, only TG and non‐TG (nTG) male mice were studied.

### 2.2 Echocardiography

**FIGURE 1:**
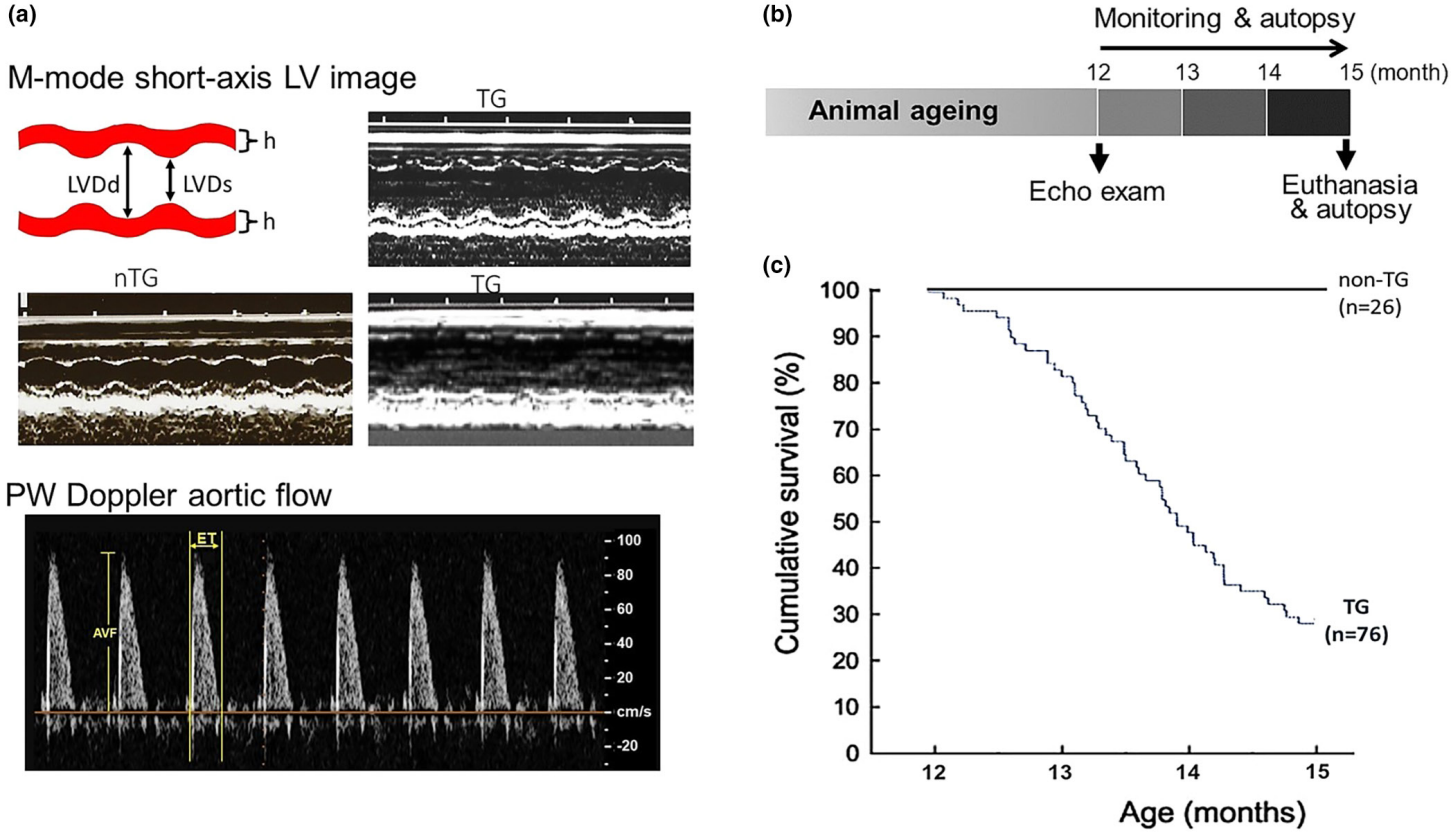
Representative echocardiographic images, study protocol, and the 15‐month survival. (a) Echocardiographic short‐axis left ventricular (LV) 2D image‐guided M‐mode traces and the Doppler aortic flow from non‐TG and TG mice. LVDd and LVDs, LV dimension at diastole and systole; h, wall thickness; AVF, aortic flow velocity; ET, ejection time. (b) Diagram depicting the study protocol. Note that all nTG and β_2_‐TG male mice entering into this study were 12 months old. (c) Kaplan–Meier survival curves of nTG and TG mice. Animals were monitored for a 3‐month period, that is, 12–15 months of age. The survival difference between nTG and TG groups was highly significant (*p* < 0.0001).

Transthoracic echocardiography was performed in 12‐month‐old mice using a Hewlett Packard Sonos 5500 ultrasound machine with a 15‐MHz linear transducer, as described previously (Gao et al., 2003; Xu et al., 2011). Mice were anesthetized by intraperitoneal injection with a mixture of ketamine/xylazine/atropine (60/12/0.6 mg/kg, respectively) and placed on a heated pad (37°C). After LV short‐axis two‐dimensional (2D) image was obtained at the papillary muscle level, 2D‐guided M‐mode traces were recorded (sweeping speed of 10 mm/s) (Figure 1a). The following parameters were measured on the M‐mode tracings using the leading‐edge technique: LV internal dimensions at diastole and systole (LVDd, LVDs), LV external dimension at diastole (exLVDd), and wall thickness at diastole (WTd). Measurements were taken from three cardiac cycles and averaged. Fractional shortening (FS) was calculated as [(LVDd–LVDs)/LVDd] × 100%. LV mass was calculated as (ExLVDd^3^–LVDd^3^) × 1.055 (1.055 is the specific gravity of muscle). Considering the features in DCM and failing heart of decline in contraction, chamber dilatation, and wall thinning, we calculated FS × (*h*/*d*) (here *h* demotes averaged anterior and posterior wall thickness at diastole, and *d* represents LVDd).

The ascending aortic flow wave was determined using pulse‐wave Doppler mode and the following parameters were determined (Figure 1b): ejection time (ET), aortic flow velocity (AFV), aortic flow velocity–time integral (VTI), and stroke volume (SV_Aorta_ = area_Aorta_ × VTI). The mean velocity of circumferential fiber shortening (Vcf) was calculated as FS/ET corrected for HR using Fridericia‐like approach [ET/(^3^√R‐R interval)], as previously described (Collins et al., 2003).

### 2.3 Animal monitoring

We previously showed in **β**_2_‐TG mice onset of DCM starts from 7 months of age and progressively worsens with aging leading to premature death (Du, Gao, Wang, et al., 2000). Male TG mice had a 12‐month survival rate of approximately 60%, and subsequent mortality was high due to HF and/or lethal arrhythmias (Du, Gao, Wang, et al., 2000; Gao et al., 2003; Nguyen et al., 2015). Accordingly, following echocardiography at 12 months of age, mice were monitored for a period of 3 months (Figure 1b), when animals were inspected twice daily to ensure the timely autopsy examination of animals found dead. On a few occasions when a mouse was found to be significantly ill (e.g., reduced physical activity, labored breath, loss of body weight or lower body temperature), a local veteran was consulted and the mouse was under close monitoring for a further 24 hours. If there was no sign of improvement or even worsening, the animal was euthanized by anesthetic overdose. Mice that survived to the age of 15 months were euthanized.

### 2.4 Morphometrics

Animals either found dead or euthanized during or at the end of the study period were autopsied. Under a surgical microscope, the chest was opened via the sternum, and the presence of pleural effusion was determined prior to isolation of the heart. The LV, RV, and atria were separated. After removing the blood clot, cardiac chambers were washed in cold saline, blotted dry, and weighed. The presence of organic thrombi in the left atrium was determined by their yellowish color and tight adhesion to atrial walls (Du, Autelitano, Dilley, et al., 2000). When atrial thrombus was identified, its weight was subtracted from heart weight. Weights of the lungs, liver, and left kidney were obtained and the tibia length (TL) was measured.

Left HF was ascertained when two of the three pathoanatomic markers were present: chronic atrial thrombus, lung weight greater than the upper limit of the nTG controls (i.e., >mean + 2SD), and pleural effusion. Numerous studies on mice with a variety of heart diseases have confirmed the close association of left HF and these markers (Du, Autelitano, Dilley, et al., 2000; Du, Gao, Wang, et al., 2000; Engelhardt et al., 1999; Ikeda et al., 2014; Liggett et al., 2000; Nguyen et al., 2019; Xu et al., 2008).

### 2.5 Histology

A mid‐portion of the LV was cut, fixed in 10% formalin in phosphate‐buffered saline, paraffin‐embedded, serially sectioned (5 μm), and stained with H&E or with Picrosirius red or Masson's trichrome. As described previously (Gao et al., 2003; Nguyen et al., 2015), histological images per LV section were randomly captured, and collagen content or cross‐sectional area (CSA) of cardiomyocytes at the level of nucleus was analyzed using the Image‐pro plus 6.0 System, without knowledge of sample grouping. From each LV section, we analyzed 6–8 histological images for collagen content, and approximately 60–80 cardiomyocytes for CSA, and the average was calculated and used. The results included mice that either died prematurely or were euthanized at the end of the study period.

### 2.6 Statistics

Results are expressed as mean ± SD or as percentages. Parameters were analyzed using one‐way ANOVA and the follow‐up analysis was done by Student t‐test if ANOVA showed between‐group difference. Ryan‐Holm step‐down Bonferroni method was used for multiple‐group comparison. Least‐square method was used for correlation and regression analysis. Kaplan–Meier curves were plotted for survival. Chi‐squared test was used for comparison of percentages between groups. *p* < 0.05 was considered statistically significant.

## 3 RESULTS

### 3.1 Animal survival

This study included 26 nTG and 76 TG male mice aged at 12 months. Following echocardiographic examination (Figure 1a), animals were followed up for 3 months (Figure 1b). At the termination date, all nTG mice survived but only 29% of TG mice were alive (Figure 1c). Of nonsurvivors, the majority of them were found dead during routine inspections and three were euthanized due to worsening of general conditions.

### 3.2 Echocardiographic findings at 12 months of age

Compared with the nTG littermates, TG mice had higher heart rates, increased LVDd and LVDs, and reduction in LV systolic function estimated by FS (Table 1). LV wall thickness was thinner at diastole while LV mass greater in TG than nTG mice. Doppler aortic flow wave measures revealed the reduction in VTI, ET, Vcf, and SV (Table 1).

**TABLE 1:** Echocardiographic parameters in 12‐month‐old nTG and β_2_‐TG mice.

Parameters	NTG	TG	TG: no HF	TG: HF	TG survivor	TG dead
n	26	76	32	44	22	54
Heart rate, bpm	423 ± 52	510 ± 94*	513 ± 72	558 ± 84*	520 ± 88	542 ± 76
ExLVDd, mm	5.7 ± 0.35	6.4 ± 0.6*	6.1 ± 0.6	6.5 ± 0.6^†^	5.8 ± 0.5	6.6 ± 0.6^†^
LVDd, mm	4.2 ± 0.4	5.0 ± 0.6*	4.8 ± 0.6	5.1 ± 0.7*	4.4 ± 0.5	5.2 ± 0.6^†^
LVDs, mm	2.7 ± 0.4	4.0 ± 0.8^†^	3.5 ± 0.7	4.2 ± 0.8^†^	3.2 ± 0.6	4.2 ± 0.7^†^
FS, %	36 ± 6	22 ± 7^#^	27 ± 8	18 ± 7^†^	28 ± 9	20 ± 7^†^
WTd, mm	0.63 ± 0.11	0.59 ± 0.13*	0.63 ± 0.12	0.56 ± 0.13*	0.61 ± 0.13	0.58 ± 0.13
*h*/*d* ratio	0.17 ± 0.04	0.13 ± 0.03^#^	0.14 ± 0.03	0.13 ± 0.03	0.15 ± 0.03	0.13 ± 0.03*
LV mass, mg	111 ± 16	142 ± 46^#^	146 ± 53	136 ± 51	141 ± 42	136 ± 47*
Aorta diameter, mm	1.58 ± 0.06	1.57 ± 0.10	1.58 ± 0.10	1.54 ± 0.11	1.56 ± 0.07	1.58 ± 0.11
AFV, cm/s	61 ± 11	57 ± 11	59 ± 11	57 ± 13	59 ± 7	58 ± 13
Ejection time, ms	59 ± 7	48 ± 7*	50 ± 7	44 ± 5^†^	51 ± 7	47 ± 6
Stroke volume, μL	49 ± 6	39 ± 10*	42 ± 11	35 ± 11*	41 ± 8	39 ± 12
VTI, mm	25.4 ± 3.4	19.6 ± 4.2*	20.6 ± 4.0	18.0 ± 4.1*	21.5 ± 3.2	19.0 ± 4.4
Vcf, circ/ETc	6.9 ± 0.9	4.5 ± 0.9*	5.5 ± 1.2	3.9 ± 1.3*	4.8 ± 1.2	4.2 ± 1.1*
FS × (h/d)	6.2 ± 1.7	3.1 ± 1.6^#^	3.6 ± 1.2	2.5 ± 1.3^†^	4.2 ± 1.6	2.6 ± 1.5^#^

*Note*: Results are mean ± SD. *p < 0.05, ^†^ *p* < 0.01, and ^#^ *p* < 0.001 versus respective nTG or TG group.

Abbreviations: AFV, aortic flow velocity; ETc, ET corrected by HR; ExLVDd, external LVD in diastole; FS, fractional shortening; h/d, WTd/LVDd ratio; LVDd, LV dimension in diastole; LVDs, LV dimension in systole; Vcf, mean velocity of circumferential fiber shortening; VTI, aortic flow velocity‐time integral; WTd, wall thickness in diastole.

### 3.3 Pathoanatomic findings

**FIGURE 2:**
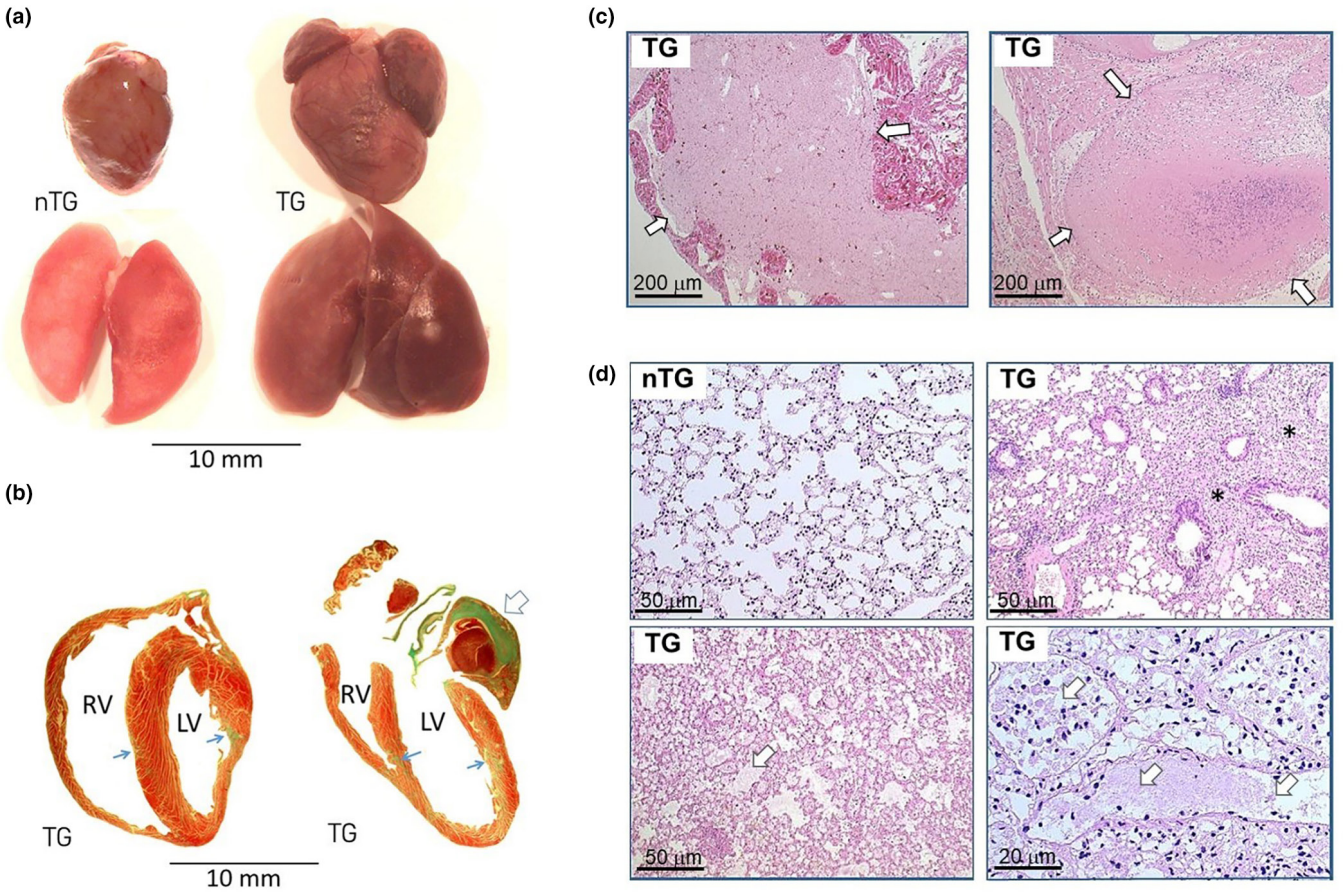
Cardiomyopathy phenotypes of β_2_‐AR transgenic mice. (a) Images of hearts and lungs of nTG and TG mice showing the enlarged heart with a thrombus in the left atrium and chronically congested lungs of TG mouse. (b) Hearts (Masson trichrome stain) from TG mice found dead of HF showing cardiomegaly, thinning of left ventricular (LV) walls, organic atrial thrombus (open arrows), patches of myocardial fibrotic scars (→), and the enlarged right ventricle (RV). (c) Histological images (H&E staining) of the enlarged left atrium containing organized thrombi (open arrows) from TG mice. (d) Histological images of lungs (H&E staining) of nTG and TG mice showing in TG mice signs indicating chronic pulmonary congestion, including thickening of alveolar walls, regional cellular proliferation (*), and accumulation of fluid and blood cells within alveolar space or microbronchial tubes (open arrows).

**FIGURE 3:**
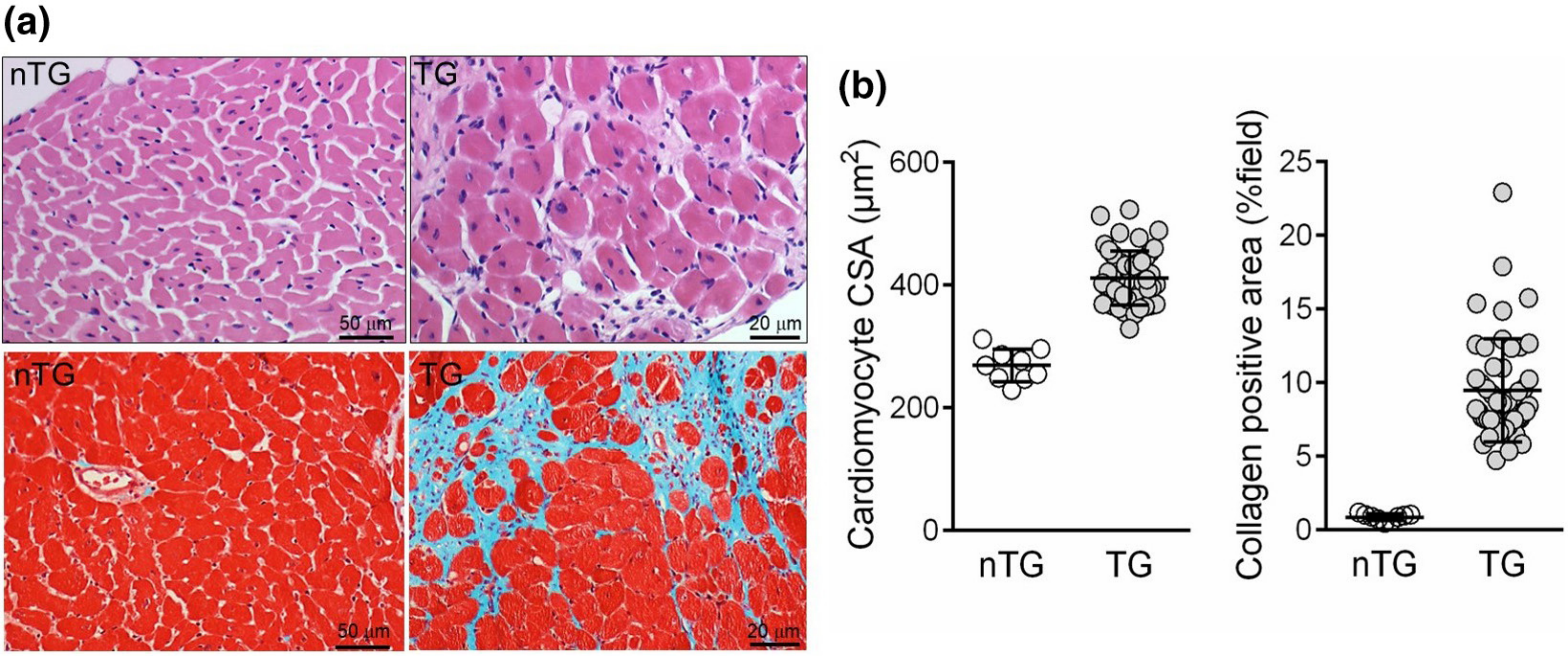
Histopathology of β_2_‐AR TG mouse hearts. (a) Histological images of the left ventricle (LV) showing cardiomyocyte hypertrophy (H&E staining) and patches or interstitial myocardial fibrosis (Masson trichrome stain). (b) Quantitative data of cardiomyocyte cross‐sectional area (CSA) or collagen‐positive stained area per view field from nTG (*n* = 9) and TG (*n* = 49) groups. Results are presented as individual data points plus mean ± SD. The between‐group differences for both parameters were highly significant (*p* < 0.0001).

All mice were autopsied. Figure 2 presents dilated LV and RV, the presence of left atrial thrombus and severe lung congestion in TG mice with HF (Figure 2a, b). The incidence of left atrial thrombus and pleural effusion was 43% and 63%, respectively (Figure 2c, Table 2), and lung weight was 60% greater in TG than nTG mice indicating pulmonary congestion (Figure 2d, Table 2). Compared with nTG littermates, TG mice had greater weights of the LV, RV, and atria leading to a 22% increase in the heart weight (Table 2). Histological analysis of the LV of TG mice showed a 50% increase in cardiomyocyte size and a 10‐fold increase in collagen‐stained area (both *p* < 0.0001, Figure 3).

**TABLE 2:** Morphometic parameters in nTG and β_2_‐TG mice at the end of study period.

Parameters	nTG	TG	TG: no HF	TG: HF	TG: Survivor	TG: Dead
*n*	26	76	32	44	22	54
BW (12‐months), g	37.0 ± 6.8	33.6 ± 5.7*	31.7 ± 4.8	35.9 ± 6.1	33.6 ± 6.1	33.9 ± 5.5
BW end, g	38.3 ± 7.7	32.2 ± 4.3*	31.8 ± 4.8	32.6 ± 4.2	32.1 ± 4.6	32.5 ± 4.1
TL, mm	18.6 ± 0.8	18.0 ± 0.5*	17.9 ± 0.5	18.1 ± 0.4	18.1 ± 0.7	18.0 ± 0.3
LV, mg	127 ± 13	148 ± 25^†^	144 ± 20	156 ± 27^†^	145 ± 32	149 ± 21
LV/BW, mg/g	3.3 ± 0.4	4.7 ± 0.8^#^	4.5 ± 0.8	4.8 ± 0.8*	4.7 ± 1.0	4.7 ± 0.7
RV, mg	28 ± 3	38 ± 10^#^	31 ± 6	44 ± 10^†^	33 ± 9	39 ± 10*
RV/BW, mg/g	0.74 ± 0.10	1.19 ± 0.34^#^	0.98 ± 0.17	1.38 ± 0.37^†^	1.07 ± 0.30	1.24 ± 0.37*
Atria, mg	14 ± 2	24 ± 10^#^	18 ± 5	29 ± 7^†^	19 ± 9	26 ± 10*
Heart, mg	170 ± 18	207 ± 32^#^	193 ± 25	224 ± 32*	200 ± 43	211 ± 31
Heart/BW, mg/g	4.5 ± 0.5	6.4 ± 1.4^#^	6.0 ± 1.0	6.8 ± 1.6*	6.1 ± 1.8	6.6 ± 1.1
Lungs, mg	177 ± 28	279 ± 54^#^	198 ± 37	358 ± 46^#^	242 ± 41	296 ± 57^†^
Lung/BW, mg/g	4.6 ± 0.8	8.7 ± 3.4^#^	6.1 ± 1.0	11.0 ± 3.0^†^	7.5 ± 2.4	9.2 ± 3.7*
Liver, g	1.77 ± 0.41	1.80 ± 0.49	1.69 ± 0.42	1.85 ± 0.41	1.55 ± 0.41	1.89 ± 0.36^#^
Liver/BW, mg/g	46.6 ± 7.0	55.3 ± 11.3*	52.6 ± 11.4	69.6 ± 10.8^†^	48.1 ± 9.9	57.8 ± 10.3^†^
Chest fluid, %	4	63^#^	16	98^#^	45	69^#^
LA thrombus, %	0	43^†^	0	77^#^	23	50^†^
Survival, %	100	29^#^	50	19^†^	—	—

*Note*: Results are mean ± SD or percentages. *p < 0.05, ^†^ *p* < 0.01, ^#^ *p* < 0.001 versus respective nTG or TG group.

Abbreviations: BW, body weight; BW‐end, BW obtained at the time when animal found dead or euthanized at the end of the study; LA, left atrium; LV, left ventricle; RV, right ventricle; TL, tibial length.

### 3.4 Correlation between echocardiographic and pathoanatomic parameters

We then analyzed correlations between 12‐month echocardiographic variables and pathoanatomic measures from mice either deceased during the follow‐up or euthanized at the end. Table 3 presents the correlation coefficient between echocardiographic measures and wet weights of the RV, atria, lungs, and liver. LV dimensions at diastole and systole and FS correlated significantly with these organ weights. Among Doppler flow‐derived parameters, ET, SV, and VTI were significantly correlated with organ weights (Table 3). Echo‐derived LV mass was correlated with the LV weight (*r* = 0.447, *p* < 0.0001). The correlations between echo variables and liver weight were poor (Table 3). FS and *h/d* ratio were negatively correlated (*r* = −0.486, *p* < 0.001).

**TABLE 3:** Coefficients of correlation between echocardiographic indices and organ weights.

	LV	RV	Atria	Lungs	Liver
FS	−0.429^#^	−0.585^#^	−0.661^#^	−0.589^#^	−0.144
LVDd	0.378^†^	0.554^#^	0.533^#^	0.467^#^	0.133
LVDs	0.376^†^	0.633^#^	0.664^#^	0.596^#^	0.162
FS×(h/d)	0.321^†^	−0.529^#^	−0.609^#^	−0.502^#^	−0.086
ExLVDd	0.438^#^	0.599^#^	0.515^#^	0.481^#^	0.174
AFV	0.219	−0.209	−0.129	−0.197	0.233
Vcf	−0.077	−0.259	−0.364^†^	−0.315^†^	−0.033
ET	−0.435^#^	−0.520^#^	−0.587^#^	−0.504^#^	−0.232
SV	−0.075	−0.470^#^	−0.342^†^	−0.412^#^	0.212
VTI	−0.165	−0.466^#^	−0.386^†^	−0.377^†^	0.116

^†^ *p* < 0.01.

^#^ *p* < 0.001.

### 3.5 Echocardiographic variables predicted HF and HF outcomes

To determine the usefulness of echocardiographic variables in predicting survival and incidence of HF during the follow‐up period, TG mice were regrouped according to the presence or absence of HF, nonsurvivor or survivor, and FS×(*h/d*), respectively. Echocardiographic variables were compared in TG mice without and with HF. As shown in Tables 1 and 2, echocardiography‐derived LV chamber size, LV mass, FS, and Vcf were significantly worse in TG mice with HF relative to non‐HF mice. TG mice with HF also had greater weights of atria, lungs, and the RV as well as poor survival versus non‐HF mice (Table 2). Comparison of TG mice of survivor or nonsurvivor groups revealed more severe LV dilatation and poor FS, higher incidence of pleural effusion and atrial thrombus, and a greater liver weight in nonsurvivor versus survivor group (Tables 1 and 2). However, Doppler flow wave‐derived variables were insignificant between HF versus non‐HF TG mice, whereas morphometric variables like LV or heart weights were comparable between survivor and nonsurvivor groups (Tables 1 and 2).

**FIGURE 4:**
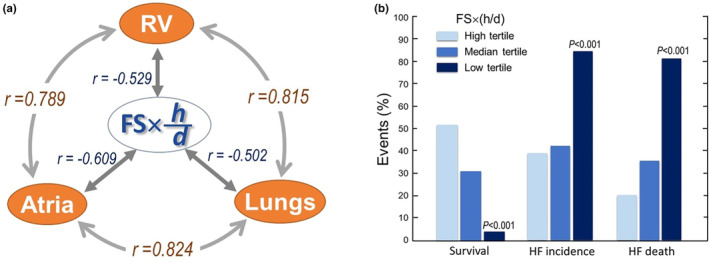
Correlations between organ weights and differences in the survival or heart failure (HF) incidence in transgenic (TG) mice grouped by fractional shortening (FS) × (*h*/*d*) tertiles. (a) Correlation coefficients depicting positive correlations between weights of atria, lungs, and the right ventricle (RV) in TG mice (*n* = 76), whereas organ weights negatively correlated with FS × (*h*/*d*) index. All correlations are highly significant (*p* < 0.001). (b) Survival or HF incidence in TG mice of FS × (*h*/*d*) tertile groups (*n* = 25–26 per group). Note that mice of the low‐tertile group had very poor survival largely due to a higher incidence of HF and HF death relative to median‐ and high‐tertile groups.

As we are aware, there has been no report on the use of echocardiographic index like FS × (*h/d*) that reflects both ventricular dysfunction and remodeling of the failing heart. The FS × (*h/d*) index ranged from 3.5 to 10 in nTG mice. In TG mice, the index was much lower and significantly correlated with wet weights of the RV, atria, and lungs (Figure 4a). Based on FS × (*h/d*), TG mice were then divided into three subgroups as high (>3.5), median (2.1–3.5), and low tertiles (<2.1). Table 4 shows that by this approach, we identified the low‐tertile group of TG mice with extremely poor survival (only 4%) and the highest HF prevalence as well as HF death (Figure 4b, Table 4). The prevalence of HF “gold” markers, pleural effusion, atrial thrombus, and pulmonary congestion was significantly higher in the low tertiles relative to the high‐tertile groups (Table 4). The liver weight was significantly greater in the low‐tertile group versus the high‐tertile group. Doppler aortic flow‐derived measures (AVF, VTI, ET, and SV) were only significantly different between low‐tertile and high‐tertile groups (data not shown).

**TABLE 4:** Comparison of pathoanatomic variables and prognosis among TG mice with different tertiles of FS × (*h*/*d*) index.

*n*	nTG	Tertiles of FS × (*h*/*d*) index			
		High (>3.5)	Median (2.1–3.5)	Low (<2.1)	
	26	25	26	25	*p*‐value
FS × (*h*/*d*)	6.2 ± 1.7	5.1 ± 1.3	2.8 ± 0.5	1.5 ± 0.4	<0.001
*h*/*d* ratio	0.17 ± 0.04	0.17 ± 0.04	0.13 ± 0.02	0.12 ± 0.02	<0.0001
FS, %	36.4 ± 6.7	30.3 ± 3.6	21.4 ± 3.9	13.0 ± 4.2	<0.001
HF incidence, %	0	40	42	84	<0.01
Survival, %	100	52	31	4	<0.01
HF death, %	0	20	35	80	<0.01
HF death/total death, %	0	42	50	87	<0.01
Pleural effusion, %	4	44	65	84	<0.01
Atrial thrombus, %	0	20	35	76	<0.01
Left ventricle, mg	127 ± 13	157 ± 30	146 ± 17	146 ± 23	<0.05
Atria, mg	14 ± 2	18.5 ± 5.6	22.2 ± 8.7	30.6 ± 8.1	<0.001
Right ventricle, mg	28 ± 3	33.9 ± 8.1	36.6 ± 12.7	42.6 ± 9.8	<0.001
Lungs, mg	177 ± 28	240 ± 41	254 ± 42	366 ± 58	<0.001
Liver, g	1.77 ± 0.41	1.69 ± 0.45	1.81 ± 0.38	1.87 ± 0.43	<0.01

*Note*: Results are mean ± SD or percentages. HF was ascertained by the presence of two of three pathoanatomical changes (atrial thrombus, pleural effusion, and pulmonary congestion). *p*‐value indicates differences among the three tertile groups of TG mice.

Abbreviations: FS, fractional shortening; *h*/*d*, WTd/LVDd ratio.

### 3.6 Pathoanatomic findings indicating compromised right heart in TG mice

The RV weight was significantly greater in TG than nTG mice, particularly in mice with signs of HF or nonsurvivor (Table 2). As shown in Figure 3b, there was evident RV enlargement and hypertrophy in mice either with left HF or died of HF. Weights of the RV, atria, and lungs were positively and significantly correlated (Figure 4a), which fits with the notion that left HF results in downstream atrial and pulmonary congestion, leading to pulmonary hypertension with subsequent RV pressure overload, hypertrophy, and dilatation. Furthermore, greater liver weight was seen in TG mice with left HF or nonsurvivors (Tables 2 and 4), indicating the onset of RV decompensation in the presence of chronic pulmonary hypertension. There was a significant although moderate degree of correlation between weights of the liver and RV (*r* = 0.303, *p* < 0.05).

## 4 DISCUSSION

In this study on aged β_2_‐TG mice with DCM, we showed that at the advanced disease stage, echocardiography is useful in predicting the HF prevalence and risk of HF death (Graphic Abstract). We found that the pathoanatomic measures were important in ascertaining the severity of cardiomyopathy and prognosis. We provided evidence to show that routine echocardiographic variables correlated well with the pathoanatomic parameters and were useful in identifying a subgroup of TG mice with high risk of congestive HF and HF death.

Previous studies implicated this TG model of DCM as a good representation of clinical ischemic or DCM leading to HF. The β_2_‐TG mice develop severe LV remodeling, dysfunction, and ventricular arrhythmias with premature deaths due to either HF or sudden death (Du, Gao, Wang, et al., 2000; Gao et al., 2003; Nguyen et al., 2015). Although there have been many reports on echocardiographic alterations in mice with cardiomyopathy, very few have linked echocardiographic changes to both morphometric measures and prognosis, including the fatality mode. In β_2_‐TG mice, the total mortality was 71% during 12–15 months of age, with 63% of fatalities attributable to HF. Despite a wide range of FS, majority of TG mice had lower FS indicating a model simulating HF with reduced EF (HFrEF) in clinical settings.

Our study revealed an extremely poor survival of male TG mice with the overall 15‐month survival of 20% considering that the mortality at 12 months of age, when the study commenced, was approximately 40% (Gao et al., 2003). Our previous studies on this TG strain of mice indicated that male TG mice at 12 months of age had severe cardiomyopathy and very high risk of premature deaths in the following few months (Gao et al., 2003). Thus, this study was commenced in TG mice from 12 months of age with a follow‐up period of 3 months. A further consideration in the study design was to generate animal data to simulate clinical patients with terminal stages of DCM, where risk stratification is critical for allocation of nondrug therapies like assistant device implantation or heart transplantation. Previous reports from this (Du, Gao, Wang, et al., 2000; Xu et al., 2008) and other groups (Engelhardt et al., 1999; Liggett et al., 2000; Tsoutsman et al., 2008) have shown that the presence of atrial thrombus, pleural effusion, increased weights of lungs, or heart chambers are reliable measures in evaluating the severity of cardiomyopathy and presence of HF. Indeed, these morphometric measures were not only useful in confirming HF but also correlated well with echocardiographic variables. Given that pathoanatomic information is rarely available in the clinic, it is desirable in preclinical studies to obtain morphometric measures for precise assessment of cardiac phenotype and to validate echocardiographic estimation of cardiac remodeling and dysfunction. Echocardiography at 12 months of age clearly revealed signs of left HF in a majority of TG mice. These TG mice that either met the criteria of left HF or found dead prior to the termination of the study had more severe cardiac phenotypes relative to other TG mice. TG mice that were found dead of non‐HF reasons were most likely due to severe ventricular tachyarrhythmias, as we previously reported (Nguyen et al., 2015). We observed in some mice while the presence of HF was evident by autopsy, animals were active by previous inspection but unexpectedly found dead by the subsequent checking. This implies coexistence of severe arrhythmias and HF, as seen in the clinical setting (Chrispin et al., 2023).

In patients with cardiomyopathy and HF, in addition to the number of comorbidities, poor prognosis is predicted based on a panel of factors including cardiac imaging‐derived LV dilatation, III–IV NYHA classification, RV dilatation, and dysfunction, as well as blood levels of biomarkers like natriuretic peptides, troponins, or catecholamines (Faggiano et al., 2021; Sinagra et al., 2020; Spinar et al., 1996; Thomas et al., 2009). Also, the presence of ventricular arrhythmias represents an independent risk of sudden death accounting for 28% of total deaths in HF patients (Cannata et al., 2020; Sinagra et al., 2020; Weintraub et al., 2017). The value of LV‐EF in prognostic prediction is less important given that the long‐term prognosis for patients with either HFpEF or HFrEF is comparable (Shah et al., 2017). Using a murine model with very poor survival during the follow‐up period, we aimed to study whether echocardiography performed at 12 months of age was useful in assessing the severity of cardiomyopathy and the overall prognosis, in particular HF death occurring in the next 3 months. We attempted to group the TG mice by the presence of HF based on the pathoanatomic criteria or by survival at the end of the study period. The two approaches of regrouping revealed that TG mice with HF or nonsurvivor had more severe LV dysfunction and LV remodeling. Despite a very low total survival of TG mice, our use of FS × (*h/d*) index allowed for identifying a cohort of mice with low tertiles and high risk of fatality and HF relative to their high‐ or median‐tertile littermates. Moreover, high‐ and low‐tertile groups differed significantly in the degree of FS reduction (by 16% and 64%, respectively, from the nTG level). Given that all β_2_‐TG mice with the same genetic background and carried identical genomic manipulation, it is interesting to observe in this model the diversity of cardiomyopathy phenotypes identified by echocardiography, morphometics, and survival monitoring, in addition to sex dimorphism as previously reported (Gao et al., 2003). Specifically, at 12 months of age, some TG mice maintained nearly normal LV contractile function despite a certain degree of chamber dilatation and hypertrophy, whereas others already degenerated into DCM and left HF. The reasons for the diversity of cardiomyopathy phenotypes in the β_2_‐TG strain remain unclear but might involve environmental, metabolic, or epigenetic factors. Further investigation is warranted to explore this fundamental question.

In patients with chronic HF, the presence of right heart dysfunction and right HF is associated with poor prognosis (Brieke & DeNofrio, 2005; Charisopoulou et al., 2019; Hiemstra et al., 2019; Kawata et al., 2017; Xu et al., 2018). Sustained elevation of pulmonary capillary pressure secondary to left HF evokes hemodynamic consequences leading to pulmonary vascular remodeling and hypertension, RV overload, and then right HF (Guazzi & Arena, 2010; Simonneau et al., 2019). Studies on mouse models of left HF have rarely attended to abnormalities of the right heart. However, in murine models of myocardial infarction or transverse aortic constriction, in which left HF is the predominant phenotype, an increase in weights of lungs, and the RV were consistently observed (Du et al., 2004; Du, Gao, Jennings, et al., 2000; Wang et al., 2000; Xu et al., 2008), indicative of RV overload and/or right HF. In aged β_2_‐TG mice, left HF is the major pathophysiological manifestation *although* ventricular arrhythmias are common (Nguyen et al., 2015). By 15 months of age, left HF was seen in approximately 55% of TG mice, and significant RV enlargement and hypertrophy were also common due to pulmonary congestion and hypertension, indicated by greater lung weight in proportion to increased RV weight. The TG mice with established left HF also had greater liver weight, likely an early sign of right HF. Increased RV and liver weights were also found in nonsurvivor versus survivor or HF versus non‐HF mice. Importantly, the severity of right heart abnormalities, estimated by changes in weights of lungs, the RV, and liver, were step‐wisely greater in high‐, median‐, and low‐tertile FS × (*h*/*d*) groups. β_2_‐TG mice had severe pulmonary congestion judged by a 130% increase in lung weights. We previously observed in a murine model of DCM with the coexistence of severe left HF and right HF, that a moderate degree of pulmonary and hepatic congestion (25%–30% increase in weights) (Nguyen et al., 2019). Such distinct differences in the severity of pulmonary congestion in both DCM models can be explained by difference in RV output in both models that determines the LV preload and hence pulmonary congestion in the setting of left HF. In future preclinical research, attention should be given to the potential abnormality of the right heart as a key component of the overall cardiac phenotype.

Several limitations deserve comment. First, the RV chamber size and function were not measured by echocardiography. Hence, the involvement of the right heart was only estimated from changes in RV weight and downstream hepatic congestion, and the presence of right HF remains speculative. Second, some important echocardiographic indices of the left heart including LV volume, LV diastolic function, and left atrial size were not determined. Third, we did not determine the severity of ventricular arrhythmias in TG mice at 12 months of age to see whether mice with frequent arrhythmic onset would more likely die of non‐HF death. Whereas β_2_‐TG mice develop frequent ventricular arrhythmias worsening with aging (Nguyen et al., 2015), arrhythmic episodes are expected to also induce hemodynamic disturbance accelerating cardiac decompensation and HF (Nguyen et al., 2017). Thus, it remains unclear why there exists diversity in the mode‐of‐death in this TG strain. Finally, female mice were not studied due to the less severe cardiomyopathy phenotype relative to male TG mice (Gao et al., 2003). Our analysis of previous acquired autopsy data from 60 female TG mice (either died or euthanized at 12–16 months of age) revealed positive correlation between RV and atria (*r* = 0.843), RV and lungs (*r* = 0.800), and atria and lungs (*r* = 0.607), respectively (all *p* < 0.0001), which were comparable to that in male TG mice.

Collectively, in the present study on aged TG male mice with advanced cardiomyopathy, HF, and poor survival, we took the approach of joint cardiac phenotyping by means of echocardiography, pathoanatomy, and survival monitoring. We showed that echocardiography‐derived parameters of LV contraction and remodeling correlated well with pathoanatomic data and are useful in identifying a subgroup of TG mice with poor prognosis particularly high HF death. Furthermore, right HF may develop in TG mice with severe left HF and a high risk of HF death.

## AUTHOR CONTRIBUTIONS

XJD conceived and designed the study. XHF, HK, ZQM, and XJD performed experiments and analyzed data. XJD drafted the manuscript and analyzed the data. HK revised the manuscript critically. All authors approved the final version of the manuscript.

## FUNDING INFORMATION

This study was supported by grants from the National Health and Medical Research Council (NHMRC) of Australia (1043026), Baker Heart and Diabetes Institute, and the Nature Science Fund of China (82070393).

## CONFLICT OF INTEREST STATEMENT

The authors have declared that no conflicts of interest exist.

## ETHICS STATEMENT

All the experimental procedures were approved by a local animal ethics committee in compliance with the Australian Code for the Care and Use of Animals for Scientific Purposes.

## Data Availability

The original data or images supporting the conclusions of this article will be made available by the authors, without undue reservation.
