# EXPRESSION OF CONCERN: Cardiomyopathy Characterizing and Heart Failure Risk Predicting by Echocardiography and Pathoanatomy in Aged Male Mice

**DOI:** 10.14814/phy2.70689

**Published:** 2025-12-03

## Abstract

**EXPRESSION OF CONCERN**: X.‐J. Du, X.‐H. Feng, Z.‐Q. Ming, and H. Kiriazis, “Cardiomyopathy Characterizing and Heart Failure Risk Predicting by Echocardiography and Pathoanatomy in Aged Male Mice,” *Physiological Reports* 12, no. 20 (2024): e70061. https://doi.org/10.14814/phy2.70061.

This Expression of Concern is for the above article, published online on October 16, 2024 in Wiley Online Library (wileyonlinelibrary.com), and has been issued by agreement between the journal Editor‐in‐Chief, Josephine C. Adams; The American Physiological Society; The Physiological Society; and Wiley Periodicals LLC. The Expression of Concern has been agreed upon after it was determined that the TG panel in figure 3a is duplicated from an article published in another journal 14 years earlier by an author common to both publications. The authors cooperated with the investigation and explained that the duplication was an inadvertent error; they also provided supporting data. However, although the conclusions are believed to remain unaffected, the journal has decided to issue an Expression of Concern to inform and alert readers of the duplication.

